# Genetic Diversity of *Pectobacterium* spp. on Potato in Serbia

**DOI:** 10.3390/microorganisms10091840

**Published:** 2022-09-15

**Authors:** Marta Loc, Dragana Milošević, Žarko Ivanović, Maja Ignjatov, Dragana Budakov, Jovana Grahovac, Mila Grahovac

**Affiliations:** 1 Department of Plant and Environmental Protection, Faculty of Agriculture, University of Novi Sad, Trg Dositeja Obradovića 8, 21000 Novi Sad, Serbia; 2 Laboratory for Seed Testing, Institute of Field and Vegetable Crops, Maksima Gorkog 30, 21101 Novi Sad, Serbia; 3 Department of Plant Diseases, Institute for Plant Protection and Environment, Teodora Drajzera 9, 11040 Belgrade, Serbia; 4 Department of Biotechnology and Pharmaceutical Engineering, Faculty of Technology, University of Novi Sad, Bulevar cara Lazara 1, 21102 Novi Sad, Serbia

**Keywords:** soft rot, blackleg, *Pectobacteriaceae*, genetic diversity, MLSA, *Pectobacterium punjabense*, potato

## Abstract

*Pectobacterium* is a diverse genus which comprises of multiple destructive bacterial species which cause soft rot/blackleg/wilt disease complex in a wide variety of crops by employing high levels of virulence factors. During the 2018, 2019 and 2020 potato growing seasons, numerous outbreaks of bacterial wilt, stem blackleg and tuber soft rot were recorded, and symptomatic plant samples from ten localities in the Province of Vojvodina (Serbia) were collected and analysed. Bacterial soft-rot pathogens were detected in 63 samples using genus and species-specific primers. Through 16S rRNA Sanger sequencing of 19 representative isolates, the identity of *P. brasiliense* (73.7%), *P. punjabense* (15.8%), and *P. carotovorum* (10.5%) species were revealed. To further validate the identification, genotypic profiling of *Pectobacterium* strains using rep-PCR (ERIC, BOX, REP) was conducted for 25 selected isolates and the phylogenetic assessment based on four selected housekeeping genes (*gyrA*, *recA*, *rpoA*, and *rpoS)*. Physiological and biochemical properties were analysed using basic microbiological tests and VITEK^®^ 2 GN card, and pathogenicity was confirmed on cv. VR808 and cv. Desiree potato tubers and plants. This study confirmed the distinctiveness of the newly described *P. punjabense* in Serbia as well as the high diversity of *Pectobacterium brasiliense* and *Pectobacterium carotovorum* species in Serbia.

## 1. Introduction

Pectinolytic bacteria from *Pectobacterium* and *Dickeya* genera are one of the most threatening phytopathogens, diverse and ubiquitous in various environments [1,2]. These opportunistic pathogens affect three of the four most important crops worldwide—rice, corn and potato, during both, production and post-harvest—processing, storage and transport [2,3]. *Pectobacterium* species are widespread in multiple ecological niches, including soil, water, weeds and potato volunteers, insects, nematodes, surfaces of contaminated field and processing equipment. They naturally colonize the surface of different plants both with and without the subsequent development of tissue maceration disease [4]. Exploiting high levels of virulence factors including plant cell wall degrading enzymes (PCWDEs), diverse regulatory systems, bacterial secretion systems, these pathogens disrupt host cell integrity and cumulatively contribute to plant collapse [5,6,7,8,9]. Considering the flexible and rapid adaptation to new hosts, wide adaptability to distinct environments and climatic conditions, these pathogens are raising serious concerns for downgrading potato yield and quality and compromising production in potato growing regions [10,11]. These destructive bacterial species cause multiple diseases of potato—wilt and upward curling of the top leaves, followed by desiccation of the whole plant, discolouration of the stem vascular tissue and external darkening [12]. In severe infections, symptoms are usually associated with soft rot of the mother tuber and rotting on the daughter tubers [13].

Latently infected seed tubers are the main inoculum source which spread through the vascular tissues colonizing and disturbing the function of the vascular system of the plant and serve to infect other plants through the root system.

Bacterial infections are mainly influenced by climate changes, higher temperatures, irregular rainfall, which cause temperature-induced shift and enhance the population development [14]. Additionally, the diversity of these species and the economic damage they cause in a specific area mainly depends on their geographic origin, trade and transport routes of planting vegetative material, the specialization of pathogens, as well as the abundance of susceptible crops. Wide scale use of advances in genomic analysis and detailed studies of the genetic diversity, provided insight into the complexity of phylogenetic relationships, established significant heterogeneity within the genus and elucidated bacterial taxonomy. The former members of Erwinia genus, with several described species and subspecies (*Erwinia carotovora* subsp. *carotovora*, *Erwinia carotovora* subsp. *atroseptica* and *Erwinia chrysanthemi*), have undergone significant taxonomic reclassification in the last few decades. Consequently, pectolytic bacteria from the genus *Erwinia* were divided into two genera-*Dickeya* and *Pectobacterium* [15,16,17]. The elevation of *Pectobacterium* subspecies to species level is progressively described and currently the total number of *Pectobacterium* species is 19-*Pectobacterium actinidiae* [18], *Pectobacterium aquaticum* [19], *Pectobacterium aroidearum* [20], *Pectobacterium atrosepticum* [16], *Pectobacterium betavasculorum* [16], *Pectobacterium brasiliense* [18], *Pectobacterium cacticida* [15,21], *Pectobacterium carotovorum* [18,22,23], *Pectobacterium fontis* [24], *Pectobacterium odoriferum* [18], *Pectobacterium parmentieri* [25], *Pectobacterium versatile* [18], *Pectobacterium wasabiae* [16], *Pectobacterium parvum* [26], *Pectobacterium polaris* [27], *Pectobacterium polonicum* [28], *Pectobacterium punjabense* [29], *Pectobacterium peruviense* [30] and *Pectobacterium zantedeschiae* [31].

*Pectobacterium* species are characterized by a broad range of distribution, wide spectrum of environments and an extremely wide range of hosts. In recent years, significant yield losses particularly on potato have constantly been reported in association with *P. atrosepticum, P. carotovorum, P. brasiliense, P. parmentieri, P. aroidearum* and the most recently described species *P. polaris, P. versatile* and *P. punjabense* [32].

In Serbia, the presence of *P. atrosepticum* and *P. carotovorum*, the causal agents of soft rot and blackleg potato, was recorded in the 1990s, after which they appeared sporadically [33,34]. *Pectobacterium brasiliense* has been characterized as the most virulent species [32], and in recent years its dominant presence has been recorded in Europe. This species has been appearing regularly since 2012 in potato production in Israel, the Netherlands, Belgium, Poland, and Switzerland [35,36,37,38,39] threatening the sustainability of potato production and reaching 20–70% of the entire population of species of this genus [32,39]. This species has also been the most commonly detected *Pectobacterium* species on potato in the past few years in Serbia [40,41,42]. The reports on the most recently described species *P. versatile* [43] and *P. punjabense* [44] also appeared in the Republic of Serbia during 2021.

This study aimed to identify the bacterial species associated with potato blackleg and soft rot registered during 2018, 2019 and 2020 potato growing seasons from ten localities of one of the main potato production regions in Serbia, the Province of Vojvodina, with particular focus on the analysis of the distinctiveness of the newly described *P. punjabense* species and diversity of *P. brasiliense* and *P. carotovorum* species. As the accurate identification becomes a very important tool for epidemiological analyses, tracking disease outbreaks and new pathogen incursions, information on the presence and prevalence of specific *Pectobacterium* species will continue to help support the management of these disease complex pathogens.

## 2. Materials and Methods

### 2.1. Sampling of Symptomatic Potato Plants and Tubers

**Figure A1:**
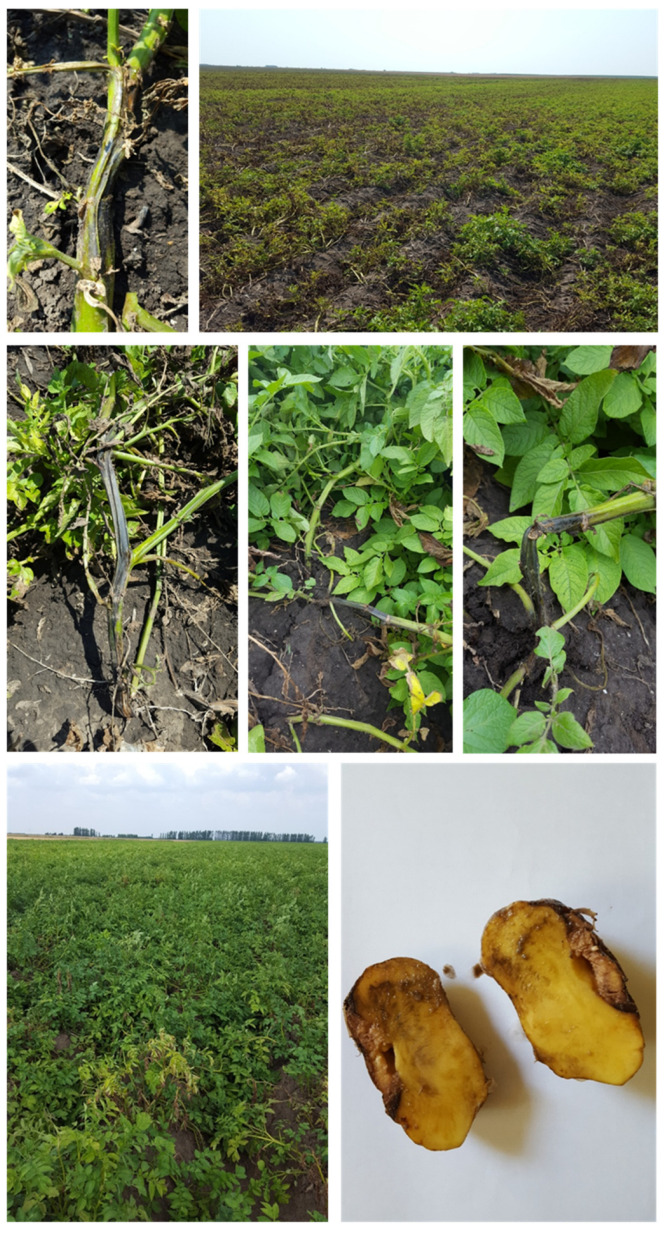
Potato blackleg, aerial stem rot, wilt, chlorosis and tuber soft rot symptoms detected in potato growing fields in the Province of Vojvodina during 2018–2020.

Potato plants with symptoms of top leaves wilting, slow growth, external darkening at the stem base and tubers softening Figure A1 (Appendix A), were collected from commercial potato fields in the Province of Vojvodina (Serbia) where potatoes are largely grown. The total number of collected samples was 86. A field inspection was conducted by moving diagonally across each field, and each sample consisted of 3 whole symptomatic plants or tubers. This was conducted on several occasions during the 2018 potato growing season from localities Obrovac (Latitude 45.349951, Longitude 19.397055), Feketić (Latitude 45.620053, Longitude 19.706665), Kula (Latitude 45.617585, Longitude 19.476082) (cv ‘Lady Claire’), during the 2019 from localities Nadalj (Latitude 45.5167087, Longitude 19.9184590), Feketić (Latitude 45.6467781, Longitude 19.6172073), Kula (Latitude 45.5986594, Longitude 19.4951055), Gradina (Latitude 45.7333226, Longitude 19.2051875), Lugovo (Latitude 45.7173731, Longitude 19.1568581), two potato plots from Maglić-Plot 28 (Latitude 45.3771370, Longitude 19.5183063), Plot 25 (Latitude 45.3713151, Longitude 19.5177893) (cv ‘Lady Claire’), two potato plots in Despotovo (Latitude 45.4616307, Longitude 19.5564352; Latitude 45.462430, Longitude 19.563750) (cv ‘Flamenco’), three potato plots in Zobnatica (Latitude 45.8500251, Longitude 19.6493442; 45.850211905081885, 19.649301282365613; 45.85110132916278, 19.65250881120089) (cv ‘VR808′), and during 2020 from Gajdobra (Latitude 45.349500, Longitude 19.385101) (cv ‘Lady Claire’). The samples were properly labelled, kept divided in polyethylene bags and transported in a portable refrigerator to the Laboratory for biological studies and pesticides of Department of Plant and Environmental Protection, Faculty of Agriculture, University of Novi Sad, Serbia, and processed immediately.

### 2.2. Bacterial Isolation

Isolation was performed from 68 potato plant and 18 tuber samples, expressing blackleg, wilting and soft rot symptoms. Bacterial strains were isolated from each sample according to standard microbiological procedures with slight modifications [45]. Plant tissue was surface sterilized with a 10% (*v*/*v*) NaOCl solution for 30 s and rinsed with sterile distilled water for 30 s and 1 cm long sections from the margins of lesions were macerated in sterile distilled water and incubated for 15 min. In order to eliminate saprophytic bacteria and to obtain preliminary pathogenicity screening, 20 µL of the homogenized macerate was transferred into a 2 mm deep cavity previously formed on 10 mm thin potato slices placed in a sterile petri dish with sterile filter paper. After 24 h of incubation, from potato slices with decay signs which served as a ‘trap’, a loopful of pure softened tissue from the margins was transferred into sterile water and incubated at room temperature (21–23 °C) for 10 min in order to allow bacteria to diffuse out from the infected tissues. Loopfulls of these homogenates were streaked onto nutrient-agar (NA) medium (Torlak, Serbia) in two replicates. Streaked NA medium plates were incubated at 26 °C for 24–48 h. Predominant shiny, cream-coloured, round colonies were obtained from all samples. A single colony of each isolate was re-streaked on NA medium and after 24 h of incubation at 26 °C, a loopful of a pure culture was suspended in 500 µL of 50% glycerol solution and stored at −80 °C.

### 2.3. Biochemical and Physiological Properties

Bacterial isolates were subjected to basic microbiological tests according to the procedures described by Schaad et al., [45], which included KOH solubility, indole, oxidase and catalase production, gelatine and nitrates reduction, tolerance to 5% NaCl, growth at 37 °C. Additionally, five selected Serbian strains and two reference strains were analysed for different carbon source utilization, enzymatic activities, and resistance using The VITEK^®^ 2 system (bioMérieux, Marcy l’Etoile, France) with Gram-Negative (GN) identification cards. The assays were performed according to the manufacturers’ instructions [46]. Firstly, the bacterial isolates were streaked on Columbia agar +5% sheep blood plates (bioMérieux, Marcy l’Etoile, France) to obtain single colonies. The bacterial suspensions were prepared from a single colony and the concentrations were checked with the VITEK colorimeter for each isolate. The GN identification card included tests for the following reactions: beta-galactosidase, beta-N-acetyl-glucosaminidase, glutamyl-arylamidase-pNAl, gamma-glutamyl-transferase, beta-glucosidase, beta-xylosidase, beta-alanine-arylamidase-pNA, alpha-glucosidase, beta-N-acetyl-galactosaminidase, alpha-galactosidase, phosphatase, glycine-arylamidase, beta-glucuronidase, glu-gly-arg-arylamidase, ala-phe-pro-arylamidase, L-pyrrolidonyl-arylamidase, L-proline-arylamidase, lipase, tyrosine-arylamidase, urease, ornithine-decarboxylase, lysine-decarboxylase, fermentation of glucose, H2S-production, and Ellman’s test. The GN card also included tests of acid production from the following substrates: sucrose, glucose, adonitol, arabitol, cellobiose, maltose, mannitol, mannose, palatinose, sorbitol, trehalose, and tagatose. Finally, the assimilation of malate, lactate, citrate, malonate, 5-keto-D-gluconate, coumarate, and histidine, as well as the alkalization of succinate and lactate, were also included.

### 2.4. DNA Extraction and PCR Detection

Prior to molecular analyses, isolates were cultured at 26 °C, and a loopful of 48 h old pure culture was transferred into 1000 µL of sterile distilled water in a 1.5 mL tube. The total DNA of the isolates was extracted using the DNeasy Plant Mini Kit (Qiagen, Hilden, Germany) according to the manufacturer’s instructions.

Isolates were subjected to PCR assay with *pel* gene-specific primers Y1 (5′-TTACCGGACGCCGAGCTGTGGCGT-3′) and Y2 (5′-CAGGAAGATGTCGTTATCGCGAGT-3′), selected from a pectate lyase-encoding gene of the Y family, which are specific for *Pectobacterium* species [47]. Moreover, PCR reaction with specific primers BR1f (5′-GCGTGCCGGGTTTATGACCT-3′) and L1r (5′-CAAGGCATCCACCGT-3′) was performed for the detection of *P. brasiliense* [48]. Oligonucleotide primers EXPCCR (5′-GCCGTAATTGCCTACCTGCTTAAG-3′) and EXPCCF (5′-GAACTTCGCACCGCCGACCTTCTA-3′) that specifically detect *P. carotovorum* [49,50,51] were also exploited in identification. Finally, the 16S rRNA PCR amplification was performed using the universal PCR primer pair 27F (5′ -AGAGTTTGATCMTGGCTCAG-3′ and 1492R (5′-TACGGYTACCTTGTTACGACTT-3′) [52]. Single PCR reactions were carried out on Surecycler 8800 Thermal Cycler (Agilent Technologies, Santa Clara, CA, USA) in a final volume of 50 μL, containing 25 µL of SuperHot MasterMix 2× (Bioron, Römerberg, Germany), 21 µL of DNA free water, 1 μL of each 10 µM primer and 2 μL of genomic DNA under the conditions presented in Table 1.

Agarose gel electrophoresis was used to determine the sizes of the amplified products with a Power station 300 (Labnet International, Inc., Corning, NY, USA) in a 1.5% agarose gel made with 1× SB (Sodium Boric Acid) Buffer and stained with ethidium bromide. GeneRuler 1 kb Plus DNA Ladder (Thermo Scientific, Waltham, MA, USA) was used as a molecular DNA marker. Electrophoresis was performed at 100 V for 35 min and the amplicons were visualized and photographed under ultraviolet (UV) light.

Production of 1460 bp amplicon of 16S rRNA was followed by Sanger sequencing (Macrogen Europe BV, Amsterdam, The Netherlands).The sequencing data was deposited in NCBI GenBank database, INSDC databases, the European Nucleotide Archive (ENA) and the DNA Data Bank of Japan (DDBJ).

Reference strains of *P. carotovorum* and *P. brasiliense* (Table 2) were included in molecular analyses for comparison.

### 2.5. DNA Fingerprinting

Rep-PCR fingerprinting was performed on the total number of 25 isolates using oligonucleotide primers ERIC1R/ERIC2, REP1R/REP2-1 and BOX-1A [54,55]. PCR reactions were performed in 25 μL, containing 12.5 µL of SuperHot MasterMix 2× (Bioron, Römerberg, Germany), 9.5 µL of DNA free water, 1 μL of 10 µM primer ERIC1R (5′-ATGTAAGCTCCTGGGGATTCAC-3′) and 1 μL of 10µM primer ERIC2 (5′-AAGTAAGTGACTGGGGTGAGCG-3′) for ERIC-PCR and REP1R (5′-IIIICGICGICATCIGGC-3′) and 1 μL of 10 µM primer REP2-1 (5′-ICGICTTATCIGGCCTAC-3′) for REP-PCR reaction, with addition of 1 μL of genomic DNA. BOX PCR reaction was performed in 25 μL, containing 12.5 µL of SuperHot MasterMix 2× (Bioron, Römerberg, Germany), 10.5 µL of DNA free water, 1 μL of 10µM primer BOX-1A (5′-CTACGGCAAGGCGACGCTGACG-3′) and 1 μL of genomic DNA.

The methods used were developed by Rademaker and de Bruijn [56], with a slight modification in the PCR conditions. PCR was performed on Surecycler 8800 Thermal Cycler (Agilent Technologies, Santa Clara, CA, USA) as follows: one cycle of 7 min at 95 °C; 35 cycles of denaturation (1 min at 94 °C), annealing (1 min at 52 °C for ERIC, at 53 °C BOX, at 40 °C REP), extension (8 min at 65 °C) and a final extension (16 min at 65 °C).

Electrophoresis was performed using 1.5% agarose gel in SB buffer at 60 V for 30 min and the next 3.5 h at 80 V. The amplicons were visualized and photographed under ultraviolet (UV) light. TriDye™ 1 kb DNA Ladder (New England Biolabs, Inc., Ipswich, MA, USA) was used as a molecular DNA marker.

The banding patterns were compared to each other and to the patterns of the reference strains and dendrograms were generated according to the unweighted pair-group mean arithmetic method (UPGMA) using PyElph version 1.3 (Python Software Foundation^®^, Wilmington, DE, USA) [57].

### 2.6. Multilocus Sequence Analysis and Phylogenetic Analysis of Pectobacterium Isolates

Six representative strains from different cluster groups based on rep-PCR, were selected for further phylogenetic analysis. Four housekeeping genes (*gyrA*, *recA*, *rpoA* and *rpoS*) were amplified with the primers gyrA1 (5′-TGGTGACGCGTCGTACCATT-3′), gyrA4 (5′-GCAGAGAACAGCATCGCTTC-3′), recA1 (5′-GGTAAAGGGTCTATCATGCG-3′), recA2c (5′-CCTTCACCATACATAATTTGGA-3′), rpoS1 (5′-ATGAGCCAAAGTACGCTGAA-3′), rpoS2 (5′-ACCTGAATCTGACGAACACG-3′) [58] and rpoA F1 (5′-GGTTCTGTGACAGAGTTTC-3′), rpoA R1 (5′-AGTTTTCCAGGCGCATGC-3′) [30]. Single PCR reactions were performed in 50 μL, containing 25 µL of SuperHot MasterMix 2× (Bioron, Römerberg, Germany), 21 µL of DNA-free water, 1 μL of each of 10 µM primer and 2 μL of genomic DNA. PCR amplification was carried out on Surecycler 8800 Thermal Cycler (Agilent Technologies, Santa Clara, CA, USA) as follows: initial denaturation (95 °C for 3 min), 32 cycles of denaturation (94 °C for 1 min), annealing (gyrA-56 °C for 1 min, recA-47 °C for 1 min, rpoS-55 °C for 1 min, rpoA-50 °C for 1 min), and extension (72 °C for 2 min), and then the final extension (72 °C for 5 min). Electrophoresis was performed at 100 V for 35 min. GeneRuler 1 kb Plus DNA Ladder (Thermo Scientific, Waltham, MA, USA) was used as a molecular DNA marker. The amplicons were visualised, photographed under ultraviolet (UV) light, and followed by Sanger sequencing (Macrogen Europe BV, Amsterdam, The Netherlands).

The gene sequences were analysed using BLASTn algorithm online at http://blast.ncbi.nlm.nih.gov/ (accessed on 25 March 2021) with already deposited sequences in NCBI GenBank database.

Sequences were merged and aligned with the *Pectobacterium* sequences retrieved from 5 genomes of members of the genus *Pectobacterium* available in GenBank (Table 3) using the ClustalW algorithm with the default settings in MEGA X software (version 10.1.7, www.megasoftware.net) (accessed on 31 March 2021) [59]. The evolutionary history was inferred by using the Maximum Likelihood method (ML) and Tamura-Nei model. The ML phylogenetic analyses were performed on individual gene sequences (*gyrA*, *recA*, *rpoA* and *rpoS*), as well as on the concatenated data for the four loci using MEGA X software. Bootstrapping was performed with 1000 replications. As an outgroup, the gene sequences of *Dickeya dianthicola* strain NCPPB 453 and *Dickeya dianthicola* strain 16LI02 were used.

### 2.7. Pathogenicity Assays

#### 2.7.1. Pathogenicity of *Pectobacterium* Isolates on Potato Tubers

Pathogenicity of all collected isolates was evaluated on potato tubers of two cultivars-cv. VR808 and cv. Desiree. Prior to the assay, potato tubers were inspected for any sign of disease or injury, washed under tap water and with sodium hypochlorite 1% (*v*/*v*), surface sterilized with ethanol 70% (*v*/*v*) and dried at room temperature over 2 h. Three potato tubers per isolate were inoculated using the toothpick piercing method [48]. Toothpicks dipped in bacterial suspension (approx. 1 × 10^8^ CFU/mL) were stabbed into the tubers. The inoculated tubers were placed into a sealed plastic container and incubated at 25 ± 2 °C. Treatment with sterile distilled water was used as a negative control. In addition to domestic Serbian isolates, trials encompassed four reference strains: *P. carotovorum* DAPP-PG 751, *P. carotovorum* DAPP-PG 752, *P. carotovorum* LMG 2408, *P. brasiliense* LMG 21370 (Table 2). The tubers were observed for soft rot symptoms for 7 days after inoculation. The pathogen was re-isolated from the rotten tubers, fulfilling Koch’s postulates and the identity was confirmed by PCR and sequencing.

#### 2.7.2. Pathogenicity of *Pectobacterium* Isolates on Potato Plant Stems

Pathogenicity test was conducted on 3-week-old healthy potato plants (cv. VR808 and cv. Desiree) grown in commercial Baltic Tray Substrate (Hawita, Vechta, Germany) in 15 cm diameter pots (Pöppelmann TEKU^®^, Lohne, Germany) under the greenhouse conditions. One potato plant stem per isolate was inoculated by the toothpick piercing method [48,60] using bacterial suspension (approx. 1 × 10^8^ CFU/mL). The test was performed in three replicates. Inoculation points 5 cm above the soil line were sealed with parafilm and were incubated under plastic bags in a greenhouse at 25 ± 2 °C. The plants inoculated with sterile toothpicks dipped in sterile distilled water served as a negative control. Reference strains (Table 2) served as a positive control. Plants were observed for blackleg symptoms for 7 days after inoculation.

## 3. Results

### 3.1. Identification and Diversity of Pectobacterium Species

Samples evaluated in this study were collected during a survey which was conducted during the 2018, 2019 and 2020 potato growing seasons from commercial potato production fields. In these years, disease incidence exceeded 40–65% in severely infected fields. The fields were monitored during July and August after the wilt, plant lodging, aerial stem rot, blackleg and tuber soft rot symptoms appeared Figure A1 (Appendix A). Lady Claire and VR808 are abundantly grown in potato fields in the Province of Vojvodina and are mainly intended for the potato chip industry. The choice of varieties for food processing, mostly potato chips, is mainly influenced by the local processing industry. However, Lady Claire was recently found to be one of the most susceptible cultivars in Serbian potato fields, considering that the most of SRP outbreaks were recorded particularly on this potato cultivar. This is supported by the fact that SPR was found on the cultivar Lady Claire at eight out of ten localities where potato plants were sampled. Similar results were recorded in the study conducted by Markovic et al., [41]. Moreover, a high incidence of SRP disease was recorded on the same cultivar in Norway in the study conducted by Rossman et al., [61]. However, commercial seed potato developers, producers and exporters do not offer information on susceptibility to SRP. Furthermore, in recent years, the newly introduced species *P. punjabese* [44] and *P. versatile* [43] previously not present in Serbia, were registered on the VR808 cultivar, probably introduced with contaminated potato planting material from the countries of Western Europe. Therefore, VR808 is also one of the most exposed cultivars, but susceptibility data is not available, neither in studies, trials, nor in the official seed potato producer’s portfolio or the European Cultivated Potato Database. Flamenco cultivar is characterized by high to very high resistance to blackleg [62], however *P. carotovorum* occurrence was registered on this cultivar in two potato plots in Despotovo, yet the occurrence was at a low level. In addition to the susceptible cultivar VR808, cultivar Desiree was also chosen for pathogenicity assay. It is a red-skinned cultivar characterized by high level of resistance to SRP [62].

A total of 86 samples were obtained for analysis; 68 potato plants with signs of wilting and stem blackleg and 18 tuber samples with symptoms of soft rot. All the bacterial cultures isolated from the samples exhibited pectolytic ability on potato slices in preliminary screening tests. The isolation and growth on NA (Nutrient Agar) from all isolates resulted in predominant shiny, transparent to cream-colored, round colonies. During this study no combined infection of different SRP species was recorded. Pure uniform colonies were restreaked on nutrient agar and the total number of 63 obtained isolates belong to the Microbial culture collection of the Laboratory for biological studies and pesticides, Department of Plant and Environmental Protection, Faculty of Agriculture, University of Novi Sad, Serbia.

### 3.2. PCR Assays

Amplification of the expected 434 bp PCR product resulted in PCR assay using Y1/Y2 primers employed to detect *Pectobacterium* spp. It was confirmed that all bacterial strains, except three strains - MMZKMVR1, MMZCVR2, and MMZKVR3, belong to the *Pectobacterium* genus. Identification using species-specific primers BR1f/L1r resulted with the expected 322 bp products and it refined the identification of *P. brasiliense* strains. The amplification of 550 bp using EXPCC primers resulted in separation of *P. carotovorum* strains.

The remaining isolates which were not detected in diagnostic PCR assays using genus and species-specific primers were evaluated using 16S rRNA amplification and sequencing (Macrogen BV, Amsterdam, The Netherlands). Moreover, to confirm the identification of the isolates which were detected using species-specific primers, isolates preliminary identified as *P. brasiliense* and *P. carotovorum*, were also included in the 16S rRNA sequencing. The 16S rRNA amplification resulted with 1420 bp products. The sequences were screened by Finch TV 1.4.0 (Geospiza, Inc., Seattle, WA, USA; www.geospiza.com) (accessed on 10 September 2019) and analysed using the BLASTn algorithm online at http://blast.ncbi.nlm.nih.gov/ (accessed on 10 September 2019).

The BLAST analyses of the 16S rRNA sequences confirmed that strains previously identified as *P. carotovorum* (MMDC11 and MMDK13) revealed 100% query coverage and 100% identity to-*Pectobacterium carotovorum* strain JB145 (Acc. No. MT579581.1), *Pectobacterium carotovorum* strain JB143 (Acc. No. MT579580.1), *Pectobacterium carotovorum* strain JR1.1 (Acc. No. CP034237.1). Sequences of Serbian strains of *P. carotovorum* were deposited in the NCBI GenBank database under the accession numbers MZ427888, MZ427897 Table A1 (Appendix B).

A comparative BLAST analysis of the 16S rRNA sequences with those retrieved from GenBank confirmed that the studied strains MMNC1, MMKC1, MMFC19, MMSGC1, MMSLC1, MMMC28, MMMC25, MMMK25, MMGKLC2, MMKC1, MMFK1, MMFC1, MMOK1 and MMOC1 belong to *P. brasiliense* regarding 100% identity with *Pectobacterium brasiliense* strain HNP201709 (Potato, South Korea), 100% identity with *Pectobacterium brasiliense* strain HNP20170 (Potato, South Korea), 100% identity with *Pectobacterium brasiliense* strain SX309 (Cucumber, China), 100% identity with *Pectobacterium brasiliense* strain IPO:4062 NAK:237 (Potato, The Netherlands), 99.93% identity with *Pectobacterium brasiliense* strain Y12 (Rape, China), 99.93% identity with Pectobacterium brasiliense strain Y12, 100% identity with *Pectobacterium brasiliense* strain HNP201709 (Potato, South Korea), 99.86% identity with *Pectobacterium brasiliense* strain BC1 (Chinese Cabbage, China), 100% identity with *Pectobacterium brasiliense* strain JB127_16s (Potato, Columbia Basin), *Pectobacterium brasiliense* strain PZ13 (Potato, South Korea), *Pectobacterium brasiliense* strain HNP201719 (Potato, South Korea), 100% identity with *Pectobacterium brasiliense* strain PYP201709 (Potato, South Korea), 100% identity with *Pectobacterium brasiliense* strain HNP201719 (Potato, South Korea), and 100% identity with *Pectobacterium brasiliense* strain HNP201719 (Potato, South Korea), respectively. The determined sequences have been deposited in the GenBank database under the accession numbers MT240621 MT240620, MT240619, MT240618, MT240617, MT240616, MT240615, MT240614, MZ427909, OM665393, OM665392, OM665394, OM665395, OM665386, respectively (Table A1 Appendix B).

Detection of *P. punjabense* was refined with 16S rRNA sequence data (GenBank Accession Numbers MZ048661, MZ048662, and MZ157274) which revealed 100% identity in addition to 100% sequence coverage of *Pectobacterium punjabense* (MT242589.1 and CP038498.1) isolated from potato in China and Pakistan, respectively. All three strains, MMZKMVR1, MMZCVR2, and MMZKVR3 were proposed to belong to *P. punjabense*.

### 3.3. Genotypic Profiling

**Figure 1:**
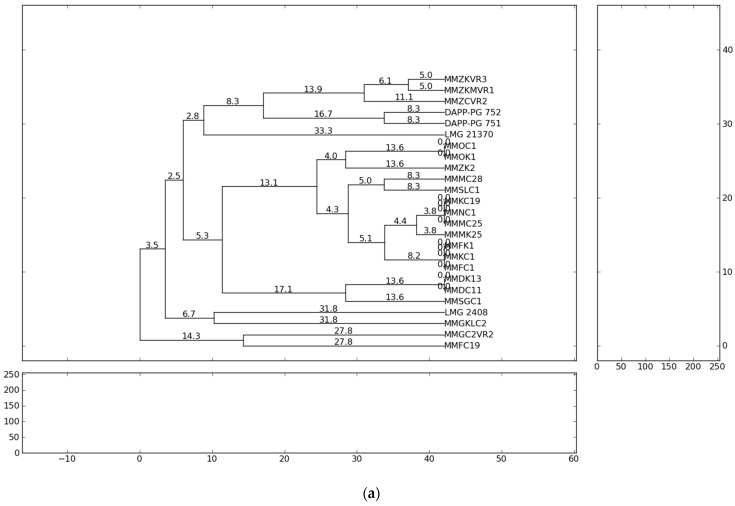

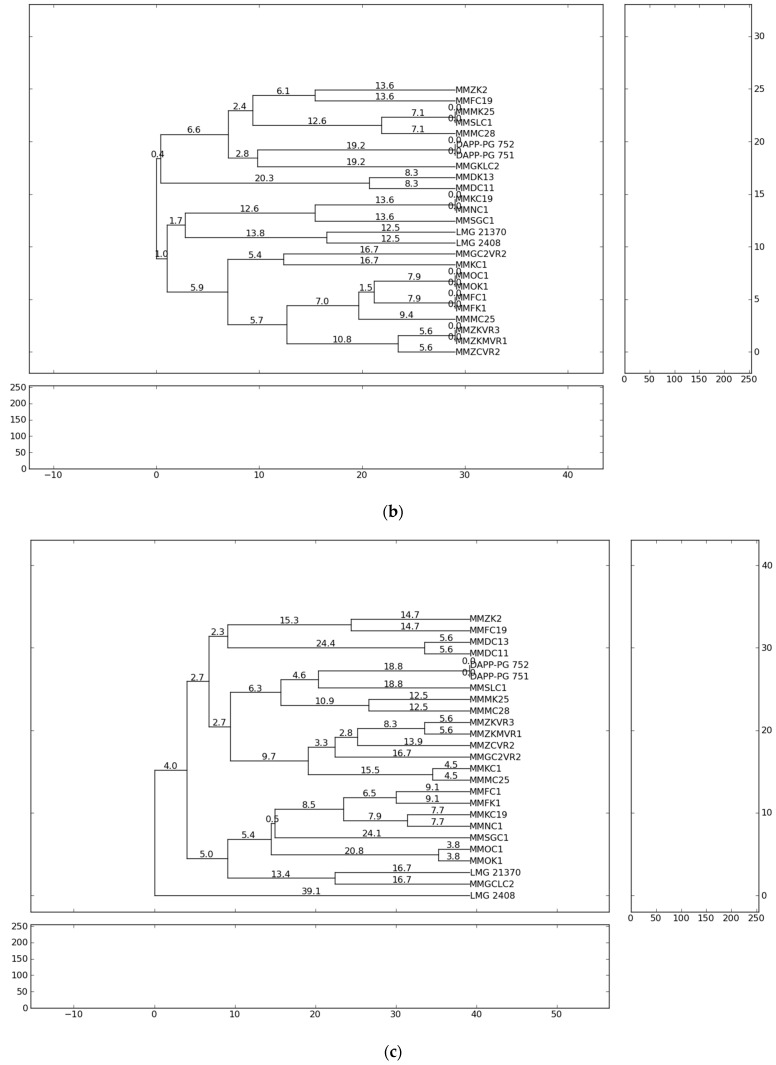
Dendrogram generated using UPGMA clustering method based on (**a**) ERIC-PCR, (**b**) BOX-PCR and (**c**) REP-PCR results for 21 isolates from Serbia and four reference strains. Genetic distances are described with numbers placed on the branches. Dendrograms were generated using PyElph version 1.3 (Python Software Foundation^®^, Wilmington, DE, USA).

The fingerprint analysis based on repetitive sequence-based PCR (rep-PCR), with primers repetitive extragenic palindromic elements (REP), enterobacterial repetitive intergenic consensus (ERIC) and BOX elements (BOX) [54,55] were used for the genetic diversity analyses of *Pectobacterium* isolates. The evaluation of genetic diversity of the 21 representative strains from Serbia, previously identified as *P. carotovorum*, *P. brasiliense* and *P. punjabense* and four reference strains originating from Lebanon, Denmark and Brazil were analysed and collectively yielded 863 bands. Independent ERIC, BOX and REP yielded 264, 276 and 323 bands, respectively. The profiles of genomic DNA fingerprinting of the *Pectobacterium* isolates showed that the number of DNA fragments generated from ERIC-, REP-, BOX-PCR varied from eight to twenty and their sizes ranged from 100 to 10,000 bp. The ERIC and BOX primers produced 11 and 13 distinct fingerprint patterns each from the 25 strains, respectively. REP-PCR produced 15 distinct banding profiles with the highest complexity in which a higher number of bands (12–20) in a range of sizes (500–10,000 bp) were obtained. The genetic relationships between the *Pectobacterium* strains evaluated on the basis of the rep-PCR fingerprints are shown in the dendrograms (Figure 1). The ERIC patterns represented amplified bands ranging from 500 to ~ 10,000 bp and grouped the strains in ten clusters, while the BOX and REP banding patterns highlighted eleven clustering groups.

The results of the present study showed that all *Pectobacterium* strains of the same species tended to group closely according to their respective taxonomic designations. The rep-PCR profiles showed high heterogeneity among isolated *P. brasiliense* strains. In summary, the present study observed the presence of high genotypic diversity among closely related strains and strains of the same species. Significant genetic difference of the strains suggests the possibility of their different geographic origin and geographic remoteness. The most probable reason for the appearance of high heterogeneity of *Pectobacterium* strains in the country could be the delivery of contaminated potato planting material from the different countries of Western Europe. However, considering that the cultivation history of the fields conducted in this study is not well-known, the infection origin can only be assumed.

### 3.4. MLSA and Phylogenetic Analysis

To further settle the taxonomic position of the obtained isolates, six selected strains from different cluster groups-four *P. brasiliense strains* MMKC19, MMFC19, MMMC28, MMSGC1, one *P. carotovorum* strain MMDC11 and one *P. punjabense strain* MMZCVR2, were selected for multilocus sequence analyses of four housekeeping genes (*gyrA*, recA, *rpoA* and *rpoS*). The comparison of the sequences analysed in this study with NCBI database sequences using BLAST*n* algorithm refined the previous identification. MMZCVR2 strain analysis showed the highest nucleotide identity (99.44 to 100%) for gyrA (Acc. No MZ161817), recA (Acc. No MZ161818), rpoA (Acc. No MZ161820) and rpoS (Acc. No MZ161821) with *P. punjabense* strain SS95 [29] previously deposited in NCBI GenBank database. Strain MMDC11 showed 99.84% to 100% identity to *P. carotovorum strain* BP201601.1 (Potato, South Korea) (99.86% gyrA, 99.84% recA, 99.86% rpoS) and WPP14 (Potato, Wisconsin, USA) (100% rpoA). Strain MMKC19 showed 100% identity to *P. brasiliense* strains: 130 (Potato, Belarus) (100% gyr A), KFB392 (Cucumber, Serbia) (100% recA), HNP201719 (Potato, South Korea) (100% rpoA), IFB5234 (Potato, Brazil) (100% rpoS). Strain MMSGC1 showed 99.68 to 100% identity to *P. brasiliense* strains: HNP201719 (Potato, South Korea) (98.19% gyrA), 1692 (Potato, Brazil) (100% recA), SX309 (Cucumber, China) (100% rpoA), strain 1009 (Potato, Germany) (99.68% rpoS). MMFC19 strain showed 98.66% homology (gyrA) and 100% homology (rpoA) with strain 130 (Potato, Belarus), 99.45% homology to strain KFB395 (Watermelon, Serbia) (recA), 99.52% to BZA12 (Cucumber, China) (rpoS). MMMC28 showed 98.33% identity with strain BZA12 (Cucumber, China) (gyrA), NJAU170 (Radish, China) (100% recA) and IFB5235 (Potato, Brazil) (100% rpoA and rpoS).

**Figure 2:**
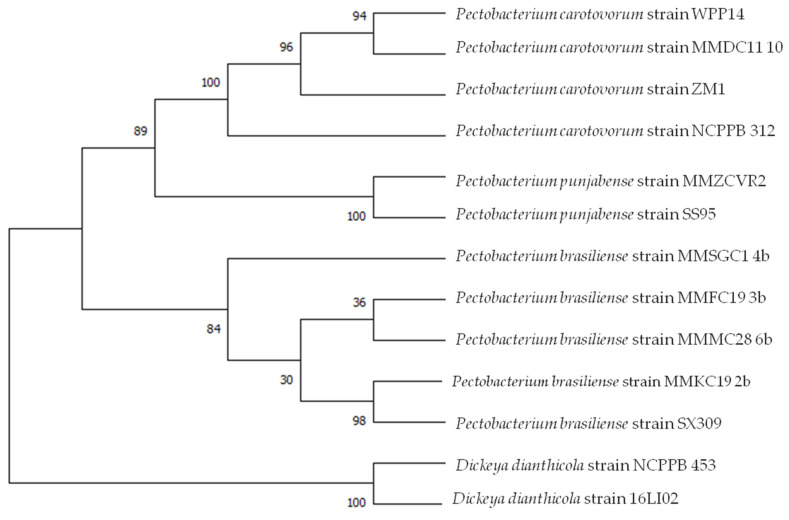
Multilocus sequence analysis of concatenated partial sequences of four housekeeping genes *gyrA*, recA, *rpoA* and *rpoS of* 13 *Pectobacterium* strains. Maximum likelihood tree built using MEGA X. *D. dianthicola* strains NCPPB 453 and 16LI02 served as an outgroup. Bootstrap values were calculated using 1000 replicates.

MLSA was carried out on the basis of sequences of selected strains from this study previously identified as *P. punjabense*, *P. brasiliense* and *P. carotovorum* and genomic data retrieved from genomes of species of the genus *Pectobacterium* available in GenBank by concatenating the nucleotide sequences of four housekeeping genes and on individual genes sequences. This analysis involved 13 nucleotide sequences. There were a total of 7441 positions in the final dataset. Evolutionary analyses were conducted in MEGA X. The evolutionary history was inferred by using the Maximum Likelihood method and Tamura-Nei model. The generated phylogenetic tree is shown in Figure 2. The tree with the highest log likelihood (−18,806.53) is shown. The percentage of trees in which the associated taxa clustered together is shown next to the branches. Initial tree(s) for the heuristic search were obtained automatically by applying Neighbor-Join and BioNJ algorithms to a matrix of pairwise distances estimated using the Maximum Composite Likelihood (MCL) approach, and then selecting the topology with superior log likelihood value. A discrete Gamma distribution was used to model evolutionary rate differences among sites (five categories (+G, parameter = 0.2546)).

MLSA supported the previous data and also highlighted that *P. punjabense* strain clustered together with the type strains SS95 (Potato, Pakistan) and clearly separated *P. punjabense* strains from closely related species. *P. carotovorum* clustered together with *P. carotovorum* strain WPP14 (Potato, WI, USA) and *P. brasiliense* strains MMFC19 and MMMC28 appeared to be highly related. Strain MMKC19 clustered together with SX309 (Cucumber, China).

The sequence data was used to calculate the average nucleotide identity (ANI) values. The number of base differences per site between sequences are shown in Table 4. Standard error estimate(s) is shown above the diagonal and was obtained by a bootstrap procedure (1000 replicates). The rate variation among sites was modelled with a gamma distribution (shape parameter = 1). This analysis involved 13 concatenated nucleotide sequences. All ambiguous positions were removed for each sequence pair (pairwise deletion option). There were a total of 7441 positions in the final dataset. Evolutionary analyses were conducted in MEGA X.

### 3.5. Biochemical and Physiological Properties

The physiological and biochemical features were used as additional methods for differentiation of *Pectobacterum* species. All of the isolates were soluble in KOH, and were therefore gram-negative. They grew at 37 °C and in 5% NaCl, reduced nitrate and gelatine. They expressed catalase activity, but did not express oxidase or acid phosphatase activity or produce indole.

**Figure 3:**
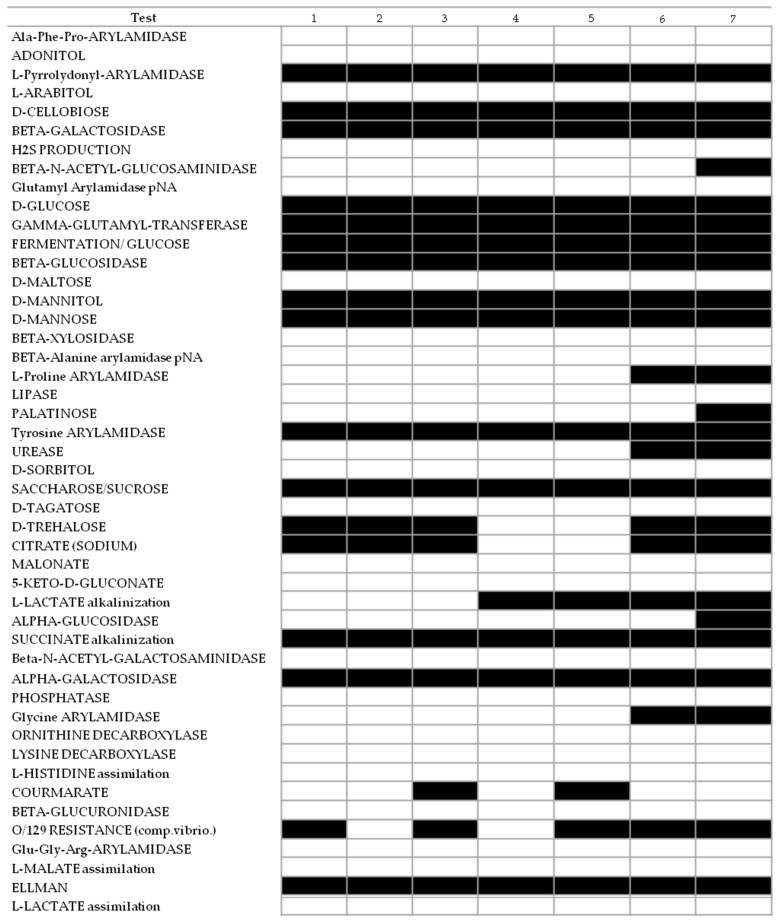
Results of the VITEK GN assay with the panel of *Pectobacterium* species. Strains 1—*P. carotovorum* strain MMDC11, 2—*P. brasiliense* strain MMKC19, 3—*P. brasiliense* strain MMSGC1, 4—*P. punjabense* strain MMZKMVR1, 5—*P. punjabense* strain MMZCVR2, 6—*P. carotovorum* strain DAPP-PG 751, 7—*P. brasiliense* strain LMG 21370.

Carbon source utilization, enzymatic activities, and resistance tests were performed for two strains of *P. punjabense*, one *P. carotovorum*, two *P. brasiliense*, and two reference strains and the results were very similar in the VITEK GN identification (Figure 3).

Enzymatic activity was negative using VITEK 2 GN cards for Ala-Phe-Pro-arylamidase, Glu-Gly-Arg arylamidase, β-glucuronidase, ornithine decarboxylase, lysine decarboxylase, 5-keto-d-gluconate and coumarate, β-alanine arylamidase pNA, β-N-acetyl-galactosaminidase, beta xylosidase, lipase. Moreover, the isolates were negative for H2S production, showed an inability for acid production from adonitole, d-sorbitole, d-tagatose, l-arabitol, l-maltose, a lack of malonate acidification and l-histidine, l-malate, and l-lactate assimilation. Contrarily, all strains were positive for L-pyrrolidonyl-arylamidase, β-galactosidase, β-glucosidase, gamma-glutamyl-transferase, tyrosine arylamidase, succinate alkanization, alpha-galactosidase, the Ellman reaction, and acid production from mannitol and mannose, sucrose, D-cellobiose, D-glucose.

*P. punjabense* strains MMZKMVR1 and MMZCVR2 were differentiated by assimilation of trehalose as the sole carbon source and sodium citrate utilization. However, reference strains displayed a biochemical pattern that was distinct for urease, L-proline arylamidase and glycine arylamidase activity. Despite the overall similarities in the biochemical profile, none of the tested isolates were identical. Among *P. punjabense* strains, slight differences, such as courmarate activity and vibriostatic agent 0/129 resistance, were recorded.

### 3.6. Pathogenicity of Pectobacterium Isolates on Tubers and Stems

**Figure 4:**
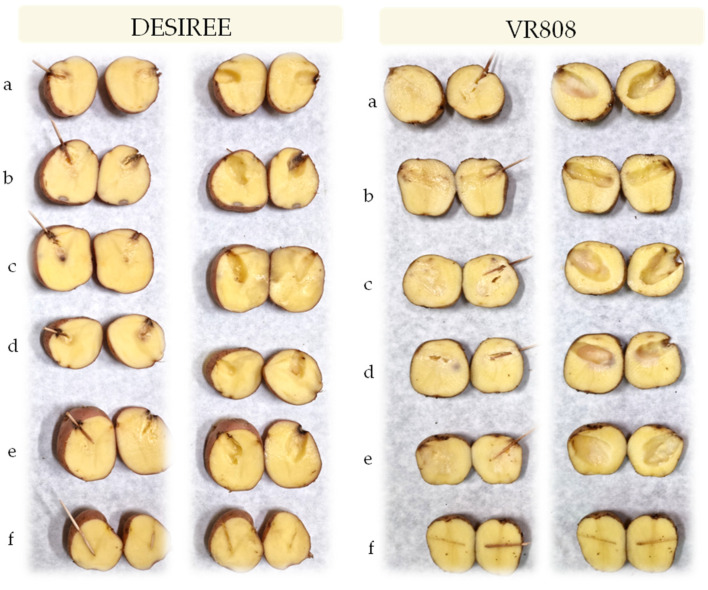
Potato tuber with soft rot registered 48 h post inoculation on cv. Desiree and VR808 inoculated with (**a**)-*P. brasiliense* strain MMKC19, (**b**)-*P. carotovorum* strain MMDC11, (**c**)-*P. punjabense* strain MMZCVR2, (**d**)-*P. carotovorum* strain DAPP-PG 752, (**e**)-*P. brasiliense* strain LMG 21370, (**f**)-negative control (left row—longitudinal section of the inoculated tuber; right row—tuber after the removal of macerated tissue).

**Figure 5:**
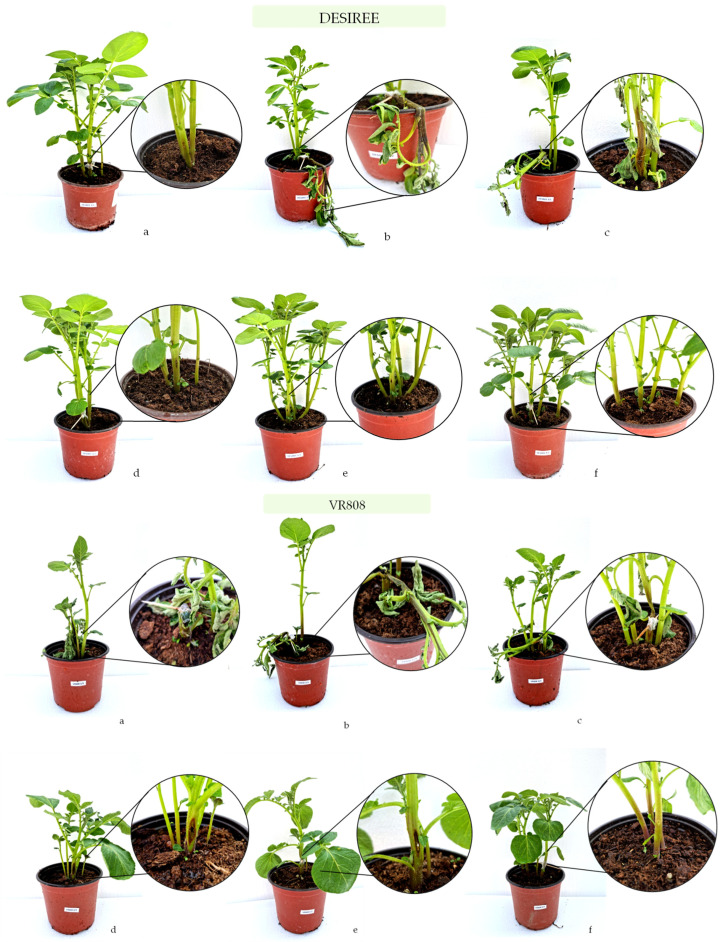
Blackleg and stem wilt symptoms registered 48 h post inoculation on cv. Desiree and cv. VR808 inoculated with (**a**)-*P. brasiliense* strain MMKC19, (**b**)-*P. carotovorum* strain MMDC11, (**c**)-*P. punjabense* strain MMZCVR2, (**d**)-*P. carotovorum* strain DAPP-PG 752, (**e**)-*P. brasiliense* strain LMG 21370, (**f**)-negative control.

In the tuber assay, all isolates caused tuber soft rot symptoms in both inoculated cultivars that were evident 48 h after inoculation and similar to those observed in the field and therefore, exhibited pectolytic ability (Figure 4). For all strains, typical blackleg or stem rotting appeared as soon as two days post inoculation (Figure 5). Four days post inoculation the plants which inoculated with all the tested strains developed symptoms of the disease and the plants completely collapsed which led to the death of the plants. The control plants treated with sterile water did not develop disease symptoms. Re-isolation, species-specific PCR and sequencing were conducted to confirm the originally isolated pathogens. The same bacteria were consistently re-isolated and identified.

## 4. Discussion

Soft rot *Pectobacteriaceae* (SRP) species have wide genetic diversity, multiple hosts and their pathogenicity is based on the production of numerous PCWDEs, including pectinases, proteases, cellulases, polygalacturonase, hemicellulases, phospholipases, xylanases, etc. [8,63,64]. In addition to exploiting the host’s rich nutrient sources, PCWDEs suppress plant defence responses and facilitate the establishment of the invading microorganism. Pectinolytic bacteria associated with potato soft rot and blackleg have recently arisen and raise serious concerns about the sustainability of potato production in the future [11].

The Republic of Serbia has areas suitable for seed and ware potato production, both in the main agricultural production areas in the Province of Vojvodina and in the mountainous region [65,66]. However, numerous *Pectobacterium* species are frequently and increasingly isolated from diseased potato originating from field and storage conditions [40,41,42,43,44]. In the highlight of recent climate changes, stressful environmental conditions that favour pathogen development-drought, followed with more frequent and heavy rainfall or floods, may be the cause of increased number of disease outbreaks and disease severity. Latently infected seeds harbouring bacterial species associated with blackleg and soft rot are the main source of inoculum and long-distance dispersal. Seed potato trade intensification is largely responsible for the wide distribution of many SRP species while their population structure may strongly reflect the import policy of a country. In countries with a more open import policy a higher diversity of SRP species is found [67]. *Pectobacterium* and *Dickeya* are regulated non-quarantine pests of potato in the European Union (Commission Implementing Regulation [EU] 2019/2072). However, *Pectobacterium* species are non-regulated pests in Serbia and *Dickeya dianthicola* is List IA Part I quarantine pests of potato seed in Serbia. Considering that potato cultivation in Serbia is mainly based on seed tubers imported from Europe, potato production is facing threats from introduction and establishment of potentially harmful pathogens previously undetected. Using a combination of physiological, biochemical and molecular diagnostic techniques, this study revealed 73.7% of *P. brasiliense* occurrence in total number of SRP species detected in this study and confirmed the increased prevalence of this newly introduced species in Serbia. *P. brasiliense* has recently been reported as one of the most commonly isolated SRP on potato in Europe [11,32,36,38,46,67,68,69,70]. This species has rapidly spread since its first appearance and has largely replaced *Dickeya* species [11,38]. In the present survey conducted during 2018–2020, no *Dickeya* species were obtained from any of soft rot and blackleg potato samples, however the presence of *Dickeya* in Serbian potato fields has recently been recorded [42]. The second most often isolated *Pectobacterium* species in this study is the rare and newly described pathogen *P. punjabense* [44] and species *P. carotovorum.*

Routine detection and identification of *Pectobacterium* spp. achieved by well-established molecular tools provide an opportunity to better understand the distribution routes and SRP diversity. Improved molecular tools provide accurate identification to the species level using primers specific to discriminative genes or genomic regions. Species-specific primers BR1f/L1r for the detection of *P. brasiliense* [48] as well as EXPCCR/EXPCCF that specifically detect *P. carotovorum* [49,50,51] used in this study offered accurate detection, as well as in numerous other studies [38,39,71,72]. However, no specific molecular tools are available to detect *P. punjabense* yet [73,74,75]. In this study, all *Pectobacterium* strains, except *P. punjabense* strains were detected in PCR assay using Y1/Y2 primers exploited to detect *Pectobacterium* spp. These *pel* gene specific primers resulted in a negative PCR and the absence of the expected 434 bp size value in case of *P. punjabense* strains. A similar result of a negative or a weak signal around the expected value was recorded in the study of Cigna et al., [73], and was explained with insufficient nucleotide specificity of Y1 primer to discriminate pectate lyase region of *P. punjabense* strains.

The 16S rRNA sequencing showed equal power of differentiation at species level, consistent with the results of MLSA. However, wide use of this gene and a large amount of reference sequences in public databases, increases the risk of misclassification. Different studies have reported very low discriminatory power which cannot guarantee species identity [15,76,77,78,79]. Moreover, some studies confirm that 16S rRNA gene resolves bacterial identification down to family or genus, however it lacks resolution below the genus level [80]. The MLSA approach can vary greatly in the selection of genes and better resolves bacterial speciation [81]. Therefore, it is a highly recommended tool for the differentiation and this is why it was employed to further validate the identification. In this study, four housekeeping genes (*gyrA*, *recA*, *rpoA* and *rpoS*), usually exploited to examine the phylogenetic relationships in SRP population were selected due to their good discriminatory value, high reproducibility, and low risk of misclassification [28,30,31]. MLSA confirmed the high diversity of *P. brasiliense* species and the identity of *P. carotovorum* and refined the identification of *P. punjabense.* This was further confirmed using maximum likelihood phylogenetic analysis which showed that the rarely occurring potato pathogen *P. punjabese* clustered closely to *P. punjabense* strain SS95.

The rep-PCR DNA fingerprinting technique is an easily accessible method that offers high throughput in comparison, targeting several repetitive elements and generating unique DNA profiles, distinguishing microbes to strain or isolate level [82,83]. *Pectobacterium* species were grouped into many different clusters by different rep-PCR analyses, indicating that high level of genetic diversity was present among the isolates, regardless of their independent geographical location or plant source specificity.

All of the obtained isolates showed pectolytic activity on potato tubers and plants, which confirmed their pathogenic character. Physiological and biochemical methods are often unable to clearly discriminate between related members of the *Pectobacterium* spp. [81]. The results of conventional biochemical tests of Serbian potato isolates mainly matched the characteristics of *Pectobacterium* sp. as previously described [16,31,84,85,86,87,88].

The monitoring and surveillance of blackleg outbreaks, sharing detection assays and exchanging of the information on the presence and prevalence of SRP species between countries are crucial to providing epidemiological insight and supporting the effective management strategies of these diseases. This study aimed to determine and characterize the population of this destructive group of pathogens in order to assess the risk of pectinolytic bacteria transitions, and to provide insight on the presence of SRP in Serbia, with the main objective to limit the damage and mitigate crop losses.

## Figures and Tables

**Table 1:** PCR conditions for specific detection of *Pectobacterium* spp., *P. brasiliense*, *P. carotovorum* and amplification of 16S rRNA.

Primer Pair	Initial Denaturation	Cycles	Denaturation	Annealing	Extension	Final Extension
**Y1/Y2**	94 °C, 10 min	25	94 °C, 60 s	67 °C, 60 s	72 °C, 30 s	72 °C, 10 min
**Br1F/L1R**	94 °C, 2 min	25	94 °C, 45 s	62 °C, 45 s	72 °C, 90 s	72 °C, 10 min
**EXPCCF/EXPCCR**	94 °C, 4 min	30	94 °C, 1 min	60 °C, 1 min	72 °C, 2 min	72 °C, 7 min
**27F/1492R**	94 °C, 5 min	30	94 °C, 30 s	53 °C, 1 min	72 °C, 30 s	72 °C, 7 min

**Table 2:** *P. carotovorum* and *P. brasiliense* reference strains used in this study.

Strain	Origin	Isolation Source	Reference
*P. carotovorum* DAPP-PG 751	Akkar, Lebanon	Potato (*Solanum tuberosum*)	-
*P. carotovorum* DAPP-PG 752	Jeb Jenin (West Bekaa), Lebanon	Potato (*Solanum tuberosum*)	[53]
*P. carotovorum* LMG 2408	Denmark	Calla Lily (*Zantedeschia aethiopica*)	[53]
*P. brasiliense* LMG 21370	Rio Grande do Sul State, Brazil	Potato (*Solanum tuberosum*)	[19,49]

**Table 3:** *Pectobacterium* genomes selected for genetic analysis.

Strain	Origin	Isolation Source	GenBank Acc. No.
*P. brasiliense* strain SX309	China	*Cucumis sativus*	CP020350.1
*P. punjabense* strain SS95	Pakistan	*Solanum tuberosum*	CP038498.1
*P. carotovorum* strain ZM1	Ukraine	-	CP045098.1
*P. carotovorum strain* WPP14	Wisconsin, USA	*Solanum tuberosum*	CP051652.1
*P. carotovorum* strain NCPPB 312	Denmark	*Solanum tuberosum*	JQHJ01000001.1
*D. dianthicola* NCPPB 453	UK	*Dianthus caryophyllus*	NZ_CM001841.1
*D. dianthicola* strain 16LI02	New York, USA	*Solanum tuberosum*	CP069602.1

**Table 4:** ANI values between related members of the genus *Pectobacterium* according to pairwise analysis (p-distance method) based on concatenated partial sequences of genes *gyrA*, recA, *rpoA* and *rpoS* between selected strains. Standard errors (SE) are shown above the diagonal and were obtained by a bootstrap procedure (1000 replicates).

SE													
	1	2	3	4	5	6	7	8	9	10	11	12	13
1. *Dickeya_dianthicola*_ strain_NCPPB_453		0.00048	0.00493	0.00683	0.00667	0.00689	0.00806	0.00487	0.00491	0.00489	0.00682	0.00784	0.00501
2. *Dickeya_dianthicola*_strain_16LI02	0.00152		0.00494	0.00681	0.00665	0.00686	0.00806	0.00488	0.00492	0.00489	0.00680	0.00784	0.00503
3. *Pectobacterium_carotovorum*_strain_NCPPB_312	0.20698	0.20667		0.00352	0.00331	0.00335	0.00366	0.00271	0.00097	0.00102	0.00183	0.00544	0.00296
4. *Pectobacterium_brasiliense*_strain_MMFC19_3b	0.14617	0.14577	0.03217		0.00213	0.00199	0.00233	0.00222	0.00366	0.00361	0.00360	0.00553	0.00515
5. *Pectobacterium_brasiliense*_strain_MMKC19_2b	0.14866	0.14828	0.03065	0.01152		0.00214	0.00261	0.00166	0.00347	0.00337	0.00352	0.00546	0.00492
6. *Pectobacterium_brasiliense*_strain_MMMC28_6b	0.13981	0.13939	0.02763	0.00842	0.01140		0.00265	0.00199	0.00341	0.00334	0.00340	0.00581	0.00514
7. *Pectobacterium_brasiliense*_strain_MMSGC1_4b	0.15443	0.15443	0.02716	0.01111	0.01289	0.01246		0.00256	0.00393	0.00389	0.00400	0.00606	0.00591
8. *Pectobacterium_brasiliense*_strain_SX309	0.20355	0.20324	0.04985	0.01221	0.00690	0.01005	0.01258		0.00274	0.00275	0.00336	0.00556	0.00339
9. *Pectobacterium_carotovorum*_strain_WPP14	0.20747	0.20716	0.00686	0.03298	0.03218	0.02805	0.02867	0.05091		0.00063	0.00067	0.00546	0.00295
10. *Pectobacterium_carotovorum*_strain_ZM1	0.20704	0.20673	0.00686	0.03298	0.03103	0.02679	0.02867	0.05124	0.00274		0.00125	0.00546	0.00294
11. *Pectobacterium_carotovorum*_strain_MMDC11_10	0.14800	0.14762	0.00930	0.03084	0.03086	0.02815	0.02862	0.02906	0.00116	0.00426		0.00548	0.00504
12. *Pectobacterium_punjabense*_strain_MMZCVR2	0.15330	0.15330	0.06972	0.07228	0.07085	0.07062	0.07564	0.07259	0.06877	0.06925	0.06828		0.00097
13. *Pectobacterium_punjabense*_strain_SS95	0.21137	0.21106	0.06390	0.07368	0.07278	0.06860	0.07949	0.08481	0.06321	0.06333	0.06931	0.00192	
p													

## Data Availability

Not applicable.

## Appendix B

**Table A1:** The list of *Pectobacterium* strains obtained in this study.

Strain	Year of Isolation	Isolation Source	Potato Cultivar	GenBank Accession Number				
				16S	gyrA	recA	rpoA	rpoS
MMNC1	2019	Potato plant	Lady Claire	MT240621	-	-	-	-
MMKC19	2019	Potato plant	Lady Claire	MT240620	MZ440812	MZ440831	MZ440821	MZ440826
MMFC19	2019	Potato plant	Lady Claire	MT240619	MZ440813	MZ440832	MZ440822	MZ440827
MMSGC1	2019	Potato plant	Lady Claire	MT240618	MZ440814	MZ440833	MZ440823	MZ440828
MMSLC1	2019	Potato plant	Lady Claire	MT240617	-	-	-	-
MMMC28	2019	Potato plant	Lady Claire	MT240616	MZ440815	MZ440834	MZ440824	MZ440829
MMMC25	2019	Potato plant	Lady Claire	MT240615	-	-	-	-
MMMK25	2019	Potato tuber	Lady Claire	MT240614	-	-	-	-
MMDC11	2019	Potato plant	Flamenco	MZ427888	MZ440816	MZ440835	MZ440825	MZ440830
MMDK13	2019	Potato plant	Flamenco	MZ427897	-	-	-	-
MMZKMVR 1	2019	Potato tuber	VR 808	MZ048661	MZ161814	-	MZ161815	-
MMZCVR 2	2019	Potato plant	VR 808	MZ048662	MZ161817	MZ161818	MZ161820	MZ161821
MMZKVR3	2019	Potato tuber	VR 808	MZ157274	-	-	-	-
MMGKLC2	2020	Potato tuber	Lady Claire	MZ427909	-	-	-	-
MMKC1	2018	Potato plant	Lady Claire	OM665393	-	-	-	-
MMFK1	2018	Potato tuber	Lady Claire	OM665392	-	-	-	
MMFC1	2018	Potato plant	Lady Claire	OM665394	-	-	-	-
MMOK1	2018	Potato tuber	Lady Claire	OM665395	-	-	-	
MMOC1	2018	Potato plant	Lady Claire	OM665386	-	-	-	
